# Comments on “Incidence of cancer among licensed commercial pilots flying North Atlantic routes”

**DOI:** 10.1186/s12940-017-0333-2

**Published:** 2017-11-17

**Authors:** S. M. J. Mortazavi

**Affiliations:** Diagnostic Imaging Center, Fox Chase Cancer Center, 333 Cottman Avenue, Philadelphia, PA 19111 USA

**Keywords:** Cancer, Pilots, Commercial flights, Cosmic radiation

## Abstract

Gudmundsdottir et al. in their paper entitled “Incidence of cancer among licensed commercial pilots flying North Atlantic routes” published in Environmental Health have evaluated the effects of exposure to higher levels of cosmic radiation on cancer incidence in the pilots of commercial flights. Despite its remarkable strengths, the paper authored by Gudmundsdottir et al. has some shortcomings. The shortcomings of this paper such as not determining the shape of dose-response relationship for radiation-induced cancers, limitations in flight dose calculations, the weaknesses of CARI-6 as the program used by Gudmundsdottir et al. to estimate the effective dose of galactic cosmic rays, and the problems associated with unpredictable nature of the magnitude and duration of solar particle events are discussed.

## Background

Pilots and aircrew are occupationally exposed to higher levels of cosmic radiation compared to our exposures on Earth [1] that is caused by decreased shielding role of the Earth’s atmosphere and the Earth’s magnetic field at flight altitudes [2]. Higher incidence of melanoma in pilots and cabin crew compared to the general population is previously reported [3]. This commentary is regarding the article by Gudmundsdottir et al. entitled “Incidence of cancer among licensed commercial pilots flying North Atlantic routes” published in Environmental Health [4]. The authors have tried to evaluate the effects of exposure to higher levels of cosmic radiation on cancer incidence in the pilots of commercial flights. The paper authored by Gudmundsdottir et al. has some shortcomings. These shortcomings and their possible effect on the validity of the findings of Gudmundsdottir et al. are discussed in detail in the following section.

## Comments

The first shortcoming of this study is due to not determining the shape of dose-response relationship for radiation-induced cancers while the authors were aware of the importance of the exposure level and cumulative radiation dose “*The RRs for all cancers, prostate cancer, melanoma, BCC, and BCC of trunk are highest in the highest exposure categories of cumulative radiation dose, and cumulative radiation dose sustained before the age of 40 years as compared with corresponding categories of duration of employment*”. It is worth noting that the shape of the dose-response curve for cancer after exposure to low doses of ionizing radiation has been a highly controversial issue for the past several decades [5]. Substantial data now indicates that in contrast to the predictions of the linear non-threshold (LNT) model; i.e. any dose of radiation, no matter how small, increases the risk of cancer [6], ionizing radiation induced-cancers may have specific thresholds of radiation dose.

Another shortcoming of this paper comes from the limitations in flight dose calculations. It is known that the factors determine the exposures of air crew are not limited to flight altitude, latitude and duration. For example, the solar activity plays a key role in this issue. Although the biological effects of the exposure of aircrew to galactic cosmic ray (GCR) are usually much larger than those of the occasional solar particle events (SPE), an intense SPE can lead to a significant rise in dose [7, 8]. While applications such as CARI-6 can be used to estimate the effective dose of GCRs, they cannot be used for estimating the effective dose received from SPEs. Despite intense SPEs are rare, the magnitude and duration of SPEs are currently unpredictable and hence they cannot be entirely ignored.

Furthermore CARI-6 unlike its updated version CARI-7A, cannot directly include heavy ion transport [9] and the effective doses calculated with CARI-7 for some flights show a significant difference (ranged from −9.2% to +23%) with those calculated with CARI-6 [10].

## Conclusion

Despite its remarkable strengths, the paper authored by Gudmundsdottir et al. has shortcomings such as not determining the shape of dose-response relationship for radiation-induced cancers, methodological problems in flight dose calculations and the limitations of CARI-6 compared to its updated version CARI-7A. These shortcomings raise questions over the validity of the findings derived.

## Abbreviations

GCR: galactic cosmic rays
LNT: linear non-threshold
SPE: solar particle events
