# Supplementary material for: Comments on “Incidence of cancer among licensed commercial pilots flying North Atlantic routes”
Source: Environ Health. 2017 Nov 17;16:125. doi: 10.1186/s12940-017-0333-2 (PMC5693704; doi:10.1186/s12940-017-0333-2)
Supplement: Related texts

# Reply: Response to comments on “Incidence of cancer among licensed pilots flying North Atlantic routes”

**Authors:** Eva Maria Gudmundsdottir, Jon Hrafnkelsson, Vilhjalmur Rafnsson

**Affiliations:** 2 0000 0004 0640 0021 grid.14013.37 Faculty of Medicine, University of Iceland, Reykjavik, Iceland; 3 0000 0000 9894 0842 grid.410540.4 Department of Oncology, Landspitali University Hospital, Reykjavik, Iceland; 4 0000 0004 0640 0021 grid.14013.37 Department of Preventive Medicine, Faculty of Medicine, University of Iceland, Reykjavik, Iceland

**Abstract**

Our study “Incidence of cancer among licensed commercial pilots flying North Atlantic routes” (4) has been commented on. We evaluated incidence of several cancer types and locations in association with cosmic radiation, and observed a positive exposure-response relation for incidence of the radiation-related cancer, basal cell carcinoma of the skin (BCC), with the four exposure metrics: employment years, cumulative air hours, cumulative radiation dose, and cumulative radiation dose sustained up to 40 years of age. The strongest relations were observed with total cumulative radiation dose and cumulative radiation dose sustained up to the age of 40 years.

**Keywords:** Basal cell carcinoma of skin, cosmic ionizing radiation, exposure-response relation

**Background**

We would like to thank dr. Mortazavi for his comment regarding our study on licensed commercial pilots (4), and at the same time we would like to clarify that we evaluated incidence of several cancer types and locations in association with gradient of cosmic radiation, and in spite of the smallness of the cohort, observed a positive exposure-response relation for incidence of the radiation-related cancer, basal cell carcinoma of the skin (BCC), with all four exposure metrics, and the strongest relation were observed with total cumulative radiation dose and cumulative radiation dose sustained up to the age of 40 years.

**Comments**

We agree with dr. Mortazavi that the use of the recently updated and improved version of the computer software CARI-7 (9) may have improved the estimation of the effective dose of ionizing radiation, and certainly we will consider that in future studies. We also agree with dr. Mortazavi that the possible biological effects of the exposure of aircrew to galactic cosmic ray are usually considered much larger than those of the solar particle events. As dr. Mortazavi says, the occurrences of solar particle events are rare, however, may be of especially short duration, and in order to take these into account in the exposure estimation in a cohort of pilots, it is necessary to have knowledge of whether each individual pilot has been flying at the time of the event, and our data do not have that precision. In our estimate of exposure metrics we had access to and used airline reports on annual number of block hours (converted to air hours), per year, per aircraft type, and per individual pilot, and that condition is of clear advantage compared with the use of retrospective self-reported work history, as has been the case in some aircrew studies.

We do not consider it appropriate, on basis of our study, to go into in-depth discussion on the predictions of the linear non-threshold model, i.e. that any small dose of radiation increase the risk of cancer, or whether ionizing radiation induced-cancers may have specific thresholds of radiation dose, but dr. Mortazavi mentions these in his commentary. In our article we frequently complained that one of the limitation of our study (4), is that the cohort is small, and the cases are not so numerous, leaving us with lack of power for complicated investigation or description of the shape of the exposure-response relation, and one of our conclusion (4) is that this relation needs to be clarified more exactly in future studies. We analysed whether the risk of cancers did become greater with gradually higher levels of exposure to cosmic radiation, where licensed pilots not employed at the airline company belonged to the lowest exposure category, and licensed pilots employed at the airline company were divided into two exposure categories, lower and higher, by the median of sustained ionizing radiation dose in milliSievert (mSv) in generalized linear model (4). In these calculations for the altogether 31 cases of BCC the relative risk (RR) became higher with higher exposure categories and the trends were statistically significant. However, according to the discussion above, we are not able to conclude on the possibility of threshold level. So in our analyses of cancer incidence in relation to the exposures we were not only evaluating “effects of exposure to higher levels of cosmic radiation on cancer incidence”, as maintained by dr. Mortazavi, but to a gradient of exposure categories (4).

In our calculations, the association of RR for BCC with ionizing radiation dose in mSv, we introduced the exposure metrics as continuous variables in the Poisson regression. In that analysis the RR was 1.0215 (95% confidence interval (CI) 1.0082 to 1.0349) for all BCC, and the RR was 1.0369 (95% CI 1.0133 to 1.0649) for BCC of trunk in association with 1 mSv increase of total cumulative radiation dose; the RR was 1.0001 (95% CI 1.0000 to 1.0002) for all BCC, and the RR was 1.0001 (95% CI 1.0000 to 1.0002) for BCC of trunk in association with 1 mSv increase of cumulative radiation dose sustained before age of 40 years. These figures are not describing the exact shape of the exposure-response relation, however, shown here for more detailed information.

**Conclusions**

In summary the most important result of our study is the strong positive exposure-response relation observed for incidence of all BCC, and BCC of trunk with the estimated total cumulative ionizing radiation dose in mSv, and cumulative ionizing radiation dose in mSv sustained up to 40 years of age. The strength of the study, as compared to previous studies on aircrews, is the use of airline reports for the calculation of the individual ionizing radiation dose, and the record linkage of the personal identifier with the comprehensive cancer registry when obtaining accurate information on the outcome, the cancer incidence. The study is the first to show that the radiation-related cancer BCC, which is not a deadly cancer, is associated with the exposure to cosmic radiation, thus the BCC among the pilots may be attributed to the cosmic radiation.
